# Epidemiology of allergic rhinitis and associated risk factors in Asia

**DOI:** 10.1186/s40413-018-0198-z

**Published:** 2018-08-06

**Authors:** Sher Ney Chong, Fook Tim Chew

**Affiliations:** Department of Biological Sciences, Faculty of Science, Allergy and Molecular Immunology Laboratory, Lee Hiok Kwee Functional Genomics Laboratories, National University of Singapore, Block S2, Level 5, Science Drive 4, Lower Kent Ridge Rd, Singapore, 117543 Singapore

**Keywords:** Allergic rhinitis, Risk factor, Asia, Systematic review

## Abstract

This review article aims to present the epidemiology and associated risk factors of allergic rhinitis (AR) in Asia. AR-related literature published on Asia was systematically reviewed and the associated risk factors were investigated.

The prevalence of AR in Asia varied considerably depending on the geographical location, study design and population involved. Several risk factors were observed to have strong association with disease presentation across multiple studies. Among these, family income, family size, daily personal computer usage time, personal and parental education attainment, and stress level have shown some level of biological gradient influence when multiple risk levels were analyzed. This suggests that AR manifestation and presentation possibly might be strongly affected by various personal and family factors. These findings are beneficial as they may provide insights into modifiable factors that may influence AR presentation. In addition, these results indicate that strategies to reduce personal and family-related risk factors have to be developed in order to alleviate the odds of AR expression.

**Electronic supplementary material:**

The online version of this article (10.1186/s40413-018-0198-z) contains supplementary material, which is available to authorized users.

## Background

### Allergic rhinitis epidemiology and symptoms

According to the Phase III International Study of Asthma and Allergies in Childhood (ISAAC), the prevalence of AR varied between 0.8 to 14.9% in 6-7 years old and 1.4 to 39.7% in 13-14 years old worldwide [1]. In Asia, this disease affects a large population, ranging from 27% in South Korea [2] to 32% in the United Arab Emirates [3].

It is a prevalent yet underappreciated atopic disorder which is commonly characterized by the presence of at least one of the following clinical symptoms: persistent nasal obstruction and mucous discharge, sneezing, and itching [4].

Although AR is commonly regarded as a mild and seasonal nuisance, it can trigger persistent mucosal inflammation which may synergize with other infective inflammation, resulting in severe outcomes including hospitalization [5]. As such, the odds of hospital admission for children with the allergic disease have been reported to increase by 19 times with the co-infection of rhinoviral diseases, allergic sensitization, and allergen exposure [6].

### Risk factors affecting the presentation of allergic rhinitis

Apart from the demographic factors, smoking and drinking habits, pet adoption, education attainment, and family history were the risk factors of AR, commonly studied in Asian countries [7–11]. Conversely, Western countries focus more on the effects of pollens, drugs, pets, and family history on the presentation of AR [12–14]. The differences between the risk factors analyzed could be culturally induced or due to the climatic differences between Asian and Western countries. However, it was observed that pet adoption and family history are the common risk factors studied in both regions, suggesting their pervasiveness in inducing AR manifestation worldwide.

### Disease diagnosis

While AR is influenced by genetic predisposition, the symptom presentation also depends on environmental exposures [15]. In addition, the disease can co-present with other diseases, such as asthma and other infectious diseases, which could further complicate the disease diagnosis. A robust association of rhinitis was found among individuals with allergic and non-allergic asthma [16]. Among patients with persistent and severe rhinitis, asthma was found prevailing [17].

Moreover, patients can experience adverse effects on social life, productivity at work and performance in school, especially for those who suffer from a more severe form of AR [16]. The use of suboptimum pharmacotherapy and antihistamines with sedative effects can further exacerbate the situation. This incurs a financial burden from both direct and indirect costs which adversely affects society [18]. Therefore, a prompt and accurate diagnosis, followed by appropriate disease management and awareness of the exacerbation risk factors, would be crucial to ease this burden.

Diagnosis of the disease is usually based on medical history of the patient in addition to skin prick test or blood test. However, misinterpretation can occasionally occur and this delays the golden treatment period which can result in other unexpected consequences, such as paying unnecessary medical expenditure and missing work [18].

### The aim of the study

This review article aims to study the epidemiology of AR in Asia and identify significant modifiable risk factors associated with disease presentation. Several criteria have been employed to establish association between triggering factors and disease manifestation.

## Methods

### Search strategy and selection criteria

The epidemiology and potential factors associated with AR manifestation were obtained from the Web of Science using the search terms of ‘rhinitis’, ‘risk’ and Asian countries. The list of Asian countries and independent territories used in the search is listed in Additional file 1. ‘Rhinitis’ is used as it represents a general form of the disease which serves to capture as many risk factors, including both modifiable and non-modifiable, as possible. As Asian and Western countries are known to have different cultural and social backgrounds, our study only evaluated articles published on Asia and this articles serves to provide a detailed list of triggering risk factors associated with AR in Asia.

Using these search terms, 56 articles were first identified. The articles were carefully reviewed and those with unclear study design or disease definition or which were conducted in a narrow pool of individuals were excluded. Apart from these 14 articles, additional 6 cross-referencing articles were also included. These 20 articles, published between 1994 and 2017, were evaluated closely for their study design, disease prevalence, disease definition, and the AR risk factor analyzed.

### Establishing the association link

The factors investigated in the 20 articles were further classified either as a potential risk factor or a co-morbidity. The association between potential modifiable risk factors and AR manifestation were evaluated using several important criteria established in literature. These criteria include the strength of association, consistency of the observed association, specificity, biologic gradient, biologic plausibility, coherence, analogy, and temporality. In addition, meta-analysis was conducted using the software/program-Stata/SE 11.2 with random effects model to evaluate the influence of modifiable risk factors with replicative results reported in at least three independent AR publications.

## Results and discussion

### AR epidemiology in Asia

Based on the methodology described, different articles published in Asia were reviewed. The reviewed articles have variable study design, disease definition and adopt different analysis parameters as shown in Table 1. The population size also varies from study to study, ranging from 200 in Kidoni et al. [19] to 30,000 in An et al. [2]

**Table 1:** Summaries of allergic rhinitis-specific articles published in year 1994-2017 in Asia

Country, location	No. of sample	Study design	Prevalence	Definition of the disease stage	Parameters analyzed	Reference, date
Singapore	2868 adults aged 20-74 years	Cross-sectional population-based study	4.5%	Allergic rhinitis: self-reported presence, in the previous year, of usual nasal blockage and discharge apart from colds or the flu, provoked by allergens, with or without conjunctivitis.	Significant parameters ➢ Age ➢ Fume exposure ➢ Housing estate ➢ Insect ➢ Occupational exposure ➢ Race ➢ Smoking Insignificant parameters ➢ Air pollution ➢ Carpet ➢ Gender ➢ Pet	Ng & Tan, 1994 [36]
Korea	10,054 residents	Cross-sectional interview based study with Physical examination	1.14%	Perennial allergic rhinitis in this study was defined as the presence of typical nasal symptoms including watery rhinorrhea, sneezing, itching and nasal obstruction during a period greater than 12 months, positive history of known allergen or triggering factors, and the physical finding of pale nasal mucosa on endoscopic examination.	Significant parameters ➢ Educational attainment ➢ Residency Insignificant parameters ➢ Marital status ➢ Occupational exposure ➢ Smoking ➢ Social class	Min et al., 1997 [20]
Thailand, Bangkok	3124 residents	Cross-sectional questionnaire based study	13.15% (95% CI = 13.13-13.17) with Chronic rhinitis (CR)	Rhinitis is defined as inflammation of the lining of the nose, characterized by one or more of the following symptoms, i.e. itching, sneezing, rhinorrhea and nasal obstruction (International Rhinitis Management Working Group, 1994). CR is diagnosed when one frequently has rhinitis symptoms without fever for a period of more than one year.	Significant parameters ➢ Associated allergic diseases ➢ Drinking ➢ Family history of atopy ➢ Household income ➢ Smoking Insignificant parameter ➢ Gender	Bunnag et al.*,* 2000 [37]
Israel	10,057 schoolchildren, aged 13-14 years	Cross-sectional questionnaire based study	41.6% with Ever AR, 9.4% with Current AR	Ever AR: Children who reported having rhinitis and sneezing without flu ever Current AR: Answer ‘Yes’ to the question, “Do you have allergic rhinitis?”	Significant parameters ➢ Asthma ➢ Family history of allergic diseases ➢ Gender ➢ Race ➢ Residency	Graif et al.*,* 2004 [38]
Singapore	202 patients aged 2-14 years	Retrospective analysis with medical records from allergic rhinitis patients undergo SPT test in KK Children’s hospital (Jul 2001 to June 2002)	33% (AR + asthma), 13% (AR + AD) & 7% (AR + asthma + AD) − 44% hospitalization rate	Confirmation from a specialist in Pediatric Otolaryngology	Significant parameter ➢ Mold	Kidoni et al., 2004 [19].
Laos, Vientiane	536 (included students aged 6-7 years and 13-14 years)	Cross-sectional questionnaire based study from Dec 2006 to Feb 2007 with stool examination	21.0% (6-7 years) & 22.3% (13-14 years)	Had a problem with sneezing, runny, or blocked nose when did not have cold or the flu in the past 12 months (ISAAC definition)	Significant parameters ➢ Household income ➢ Parasitic infection ➢ Past respiratory infection Insignificant parameters ➢ Age ➢ Air conditioning ➢ Birth order ➢ Family history of allergic diseases ➢ Food ➢ Gender ➢ Parity ➢ Past measles infection ➢ Pet ➢ Sharing bed ➢ Smoking ➢ Time on road	Phathammavong et al.*,* 2008 [9]
Singapore	6794 children attending 120 randomly selected child care centres	Cross-sectional questionnaire based study	25.6 (Rhinitis)	N.A.	Significant parameter ➢ Smoking	Zuraimi et al., 2008 [39]
Taiwan, Taipei	1368 elementary school children	Cross-sectional questionnaire based study with multi-stage clustered-stratified random method, physical examination	50.1%	The presence of typical nasal symptoms including watery rhinorrhea, sneezing, and nasal obstruction of more than 12 months’ duration, positive history of known allergen or triggering factors, and pale nasal mucosa.	Significant parameters ➢ Air pollution ➢ Carpet ➢ Gender ➢ Parity Insignificant parameters ➢ Age of gestation ➢ Gestational complication ➢ Maternal education ➢ Mold ➢ Pet ➢ Smoking	Hsu et al.*,* 2009 [10]
United Arab Emirates, Al-Ain City	7550 residents ≥13 years	Cross-sectional questionnaire based study	32%	The definition of AR used in this study was having had AR symptoms of (nasal blockage, rhinorrhoea, sneezing and irritation), in the past 12 months.	Significant parameters ➢ Age ➢ Education attainment ➢ Family history of allergic diseases ➢ Gender ➢ Nationality	Alsowaidi et al., 2010 [3]
Singapore	2994 children living in homes without any indoor risk factors	Cross-sectional questionnaire based study	24% (Rhinitis)	N.A.	Significant parameter ➢ Traffic Insignificant parameter ➢ Air conditioning	Zuraimi et al.*,* 2011 [21]
China, Guangzhou City	9899 citizens	Cross-sectional questionnaire based study with stratified multistage cluster sampling method	6.24%	According to the diagnostic criteria of AR in the ARIA 2001 Guideline, the ENT specialists verified the screening questionnaires and made the diagnosis based on the typical AR symptoms within the last 12 months. Intermittent AR was determined when the symptoms occur, 4 days/week or, 4 consecutive weeks/year; while persistent AR was determined when symptoms last 4 days/week or 4 consecutive weeks/year.	Significant parameters ➢ Computer usage ➢ Family history of allergic diseases ➢ Home renovation ➢ Pet ➢ Residency ➢ Smoking Insignificant parameters ➢ Age ➢ Breastfeeding ➢ Car ownership ➢ Hair coloring ➢ Household income	Li et al.*,* 2014 [7]
Korea	31,217 subjects aged 6-97 years	Cross-sectional study, data from Korea National Health and Nutrition Examination Survey	27%	N.A.	Significant parameters ➢ Marital status ➢ Occupational exposure ➢ Sleep time ➢ Stress level Insignificant parameters ➢ BMI ➢ Drinking ➢ Education attainment ➢ Family size ➢ Household income ➢ Residency ➢ Smoking	An et al., 2015 [2]
China	20,803 elementary school students	Cross-sectional questionnaire based study	9.8%	AR: yes for “Has your child had allergic rhinitis in the past 12 months?”	Significant parameters ➢ Age ➢ Age of gestation ➢ Breastfeeding ➢ Family size ➢ Gender ➢ Household income ➢ Housing estate ➢ Maternal education ➢ Mode of delivery ➢ Maternal pre- or postnatal depression ➢ Paternal education Insignificant parameters ➢ Drinking ➢ Smoking	Li et al.*,* 2015 [22]
Malaysia	695 Malaysia office works aged 18-60 years	Cross-sectional questionnaire based study, SPT test, building inspection	53% with current rhinitis	Doctor diagnosis	Significant parameters ➢ Age ➢ House dust mite Insignificant parameters ➢ Gender ➢ Pet ➢ Smoking	Lim et al.*,* 2015 [11]
China, Wuhan	3327	Cross-sectional questionnaire based study, physical examination	17.67%	Doctor diagnosis	Significant parameter ➢ Gender Insignificant parameter ➢ BMI	Lei, Yang & Zhen, 2016 [40]
Malaysia, Johor Bahru	462 students from 8 random schools	Cross-sectional questionnaire based study, building inspections	18.8% for students from junior high schools	N.A.	Significant parameter ➢ Fungi	Norbäck et al.*,* 2016 (1) [41]
Malaysia, Johor Bahru	462 students from 8 random schools	Cross-sectional questionnaire based study, building inspections	18.8% for students from junior high schools	N.A.	Significant parameters ➢ Atopy ➢ Family history of allergic disease ➢ Fungi ➢ House dust mite ➢ Race Insignificant parameters ➢ Gender ➢ Smoking	Norbäck et al.*,* 2016 (2) [35]
China, Shanghai	13,335 children, aged 4-6 years	Cross-sectional questionnaire based study	12.6%	Answer yes for “Has your child ever had a problem with sneezing, or a runny, or blocked nose when he/she did not have a cold or the flu in the past years”	Significant parameters ➢ Breastfeeding ➢ Gruel introduction	Huang et al.*,* 2017 [34]
Taiwan	1497 newborns	Birth cohort follow-up, questionnaire survey, physician-verified and serological testing	Non-atopic parents & one atopic parent & atopic parents : 30.8% vs 39.9% vs 54.7%	Doctor diagnosis	Significant parameters ➢ Age of gestation ➢ Gender ➢ Residency	Lee et al.*,* 2017 [42]
Kuwait	1154 students, aged 18-26 years attending Kuwait University	Cross-sectional questionnaire based study	20.4% (95% Cl- 18.1-22.9)	Current rhinitis: “ever doctor-diagnosed rhinitis” plus “having problems with sneezing, runny, or blocked nose in the absence of cold or flu in the last 12 months”	Significant parameters ➢ Age ➢ Family history of allergic diseases ➢ Pet Insignificant parameters ➢ Birth order ➢ Gender ➢ Mode of delivery ➢ Smoking	Ziyab, 2017 [8]

Though similar parameters were used to study the epidemiology of AR, a larger population group will help to further establish the prevalence of the disease as it better represents the targeted population. In addition, apart from the country of study, the disease prevalence differs depending on the disease definition and the study population. In the study conducted by Min et al. [20], AR prevalence is 1.14% among Korean residents; while in a retrospective study published by Alsowaidi et al., 2010 [3], 32% of United Arab Emirates residents are AR patients.

### Risk factors and co-morbidities of AR

Apart from the general demographic factors, many modifiable risk factors for allergic diseases, such as smoking and drinking habits were investigated as summarised in Table 1. Furthermore, cultural- or socioeconomic-related factors specific to an individual country have been explored in some studies to identify their association with AR presentation. For instance, heavy traffic and individual stress level are two factors investigated in a Singapore [21] and Korea [2] study, respectively. These factors were identified worrying elements in the respective countries, thus finding their association with AR presentation is crucial.

We further classified these factors into a potential risk factors or co-morbidities category based on the following definitions. A typical risk factor is a demographical, physical, sociological or environmental component which potentially increases the risk of presenting a disease or is protective against the expression of an illness. On the other hand, if AR manifestation is linked to another disease occurrence, it will be known as a co-morbid of AR. As listed in Table 2, most of the factors analysed are in the risk factor category. However, diseases such as asthma are co-morbidities which can possibly induce AR expression as shown in Table 3. In this article, only the modifiable risk factors were evaluated for their relationship with AR manifestation.

**Table 2:** The list of risk factors analyzed in the literature reviewed

No.	Risk Factors
1	Age
2	Age of gestation
3	Air conditioning
4	Air pollution
5	Alcohol consumption (self/parent)
6	Birth order
7	BMI
8	Breastfeeding
9	Car ownership
10	Carpet
11	Computer usage
12	Drinking (self/parent)
13	Education attainment
14	Family history of allergic diseases
15	Family history of atopy
16	Family size
17	Food
18	Fume exposure
19	Fungi
20	Gender
21	Gestational complication
22	Gruel introduction period
23	Hair coloring
24	Home renovation
25	House dust mite
26	Household income
27	Housing estate
28	Insect
29	Marital status
30	Maternal education
31	Maternal pre- or postnatal depression
32	Mode of delivery
33	Mold
34	Nationality
35	Occupational exposure
36	Parasitic infection
37	Parity
38	Past measles infection
39	Past respiratory infection
40	Paternal education
41	Pet
42	Race
43	Residency
44	Sharing bed
45	Sleep time
46	Smoking (self/parent)
47	Social class
48	Stress level
49	Time on road
50	Traffic

**Table 3:** The list of co-morbidities analyzed in the literature reviewed

No.	Co-morbidities
1	Atopy
2	Associated allergy
3	Current asthma

### Demographical factors affecting the AR presentation

Multiple papers have suggested the importance of age, gender, race, and nationality in affecting AR presentation (Table 4). The association of race and nationality on the disease expression could signify the difference in social and cultural backgrounds, as well as genetics, which can potentially influence the presentation of AR. However, as these factors are non-modifiable, they are only useful in evaluating the risk of presenting AR, but not for prevention.

**Table 4:** Strength of association of demographic factors with AR manifestation

Study	Study population, N	OR/PR^a^	Values (95% CI)	*p*-value	References
Age					
Alsowaidiet al., 2010 [3]	7550	OR	0.66 (0.54 - 0.81)	< 0.0005	> 19 years in ref. to 13-19 years: OR adjusted for nationality, gender, family history of AR, and education
Li et al., 2015 [22]	20,803	OR	1.05 (1.02-1.07)	< 0.05	Continuous variable, 1 year increase (elementary school student)
Lim et al.*,* 2015 [11]	695	OR	0.72 (0.58 - 0.88)	< 0.01	Continuous variable, 10 year increase (18 - 60 years): OR adjusted for gender, smoking, house dust mite allergy, cat allergy, home dampness, and home renovation
Ng & Tan, 1994 [36]	2868	OR	0.19 (0.10 – 0.35)	< 0.0001	60-74 years in ref. to 20-39 years: OR adjusted for race, flat size, housing estate, smoking, insect exposure, occupational exposure, and fume
Ziyab, 2017 [8]	1154	PR	1.04 (1.01 - 1.07)	< 0.01	Continuous variable (18-26 years): PR adjusted for gender, cat exposure, maternal AR, and paternal AR
Gender					
Alsowaidi et al., 2010 [3]	7550	OR	0.75 (0.63 - 0.88)	< 0.005	Male in ref. to female: OR adjusted for nationality, age, family history of AR, and education
Graif et al.*,* 2004 [38]	10,057	OR	0.85 (0.74 – 0.97)	–	Male in ref. to female: OR adjusted for current asthma, family history of asthma, race, residency, and smoking
Hsu et al., 2009 [10]	1368	OR	0.58 (0.47 – 0.72)	< 0.001	Male in ref. to female: OR adjusted for birth weight, parity, gestational age, maternal education, gestational complications, smoking, pets, carpets, molds, and air pollutions
Lee et al., 2017 [42]	1497	OR	1.57	< 0.01	Male in ref. to female
Lei, Yang & Zhen, 2016 [40]	3327	OR	0.68 (0.46 - 1.00)	< 0.05	Male in ref. to female
Li et al.*,* 2015 [22]	20,803	OR	1.55 (1.41 - 1.70)	< 0.001	Male in ref. to female
Race					
Graif et al.*,* 2004 [38]	10,057	OR	1.75 (1.45 - 2.13)	–	Jews in ref. to Arabs: OR adjusted for current asthma, family history of asthma, gender, residency, and smoking
Ng & Tan, 1994 [36]	2, 868	OR	2.02 (1.29 - 3.14)	< 0.005	Indian in ref. to Malay: OR adjusted for age, flat size, housing estate, smoking, insect exposure, occupational exposure, and fume
Norbäck et al.*,* 2016 (2) [35]	462	OR	0.33 (0.13 - 0.88)	< 0.05	Indian in ref. to Malay: OR adjusted for gender, smoking, atopy, and family history of allergic diseases
Nationality					
Alsowaidi et al.*,* 2010 [3]	7550	OR	0.48 (0.34 - 0.68)	< 0.005	Others in ref. to Arabs: OR adjusted for age, gender, family history of AR, and education
Residency					
Graif et al.*,* 2004 [38]	10,057	OR	0.84 (0.90 - 1.40)	–	Urban in ref. to rural: OR adjusted for current asthma, family history of asthma, gender, gender, and smoking
Lee et al., 2017 [42]	1497	OR	0.71	< 0.05	Townhouse in ref. to others
Li et al.*,* 2014 [7]	9899	OR	1.91 (1.37 - 2.68)	< 0.001	Urban in ref. to rural
Min et al., 1997 [20]	10,054	OR	5.26 (2.27 - 12.50)	< 0.05	Urban in ref. to rural: OR adjusted for age
Housing estate					
Li et al.*,* 2015 [22]	20,803	OR	2.19 (1.97 - 2.43)	< 0.001	Cities SH, GZ, WH, CD in ref. to XA, HA, HO, UR
Ng & Tan, 1994 [36]	2868	OR	1.92 (1.07 - 3.46)	< 0.05	Toa Payoh in ref. to Yishun : OR adjusted for age, flat size, race, smoking, insect exposure, occupational exposure, and fume
Household income					
Bunnag et al.*,* 2000 [37]	3124	OR	1.97 (1.23 - 3.16)	< 0.05	High income in ref. to medium income: adjusted OR
Li et al., 2015 [22]	20,803	OR	1.42 (1.21 - 1.68)	< 0.001	800-1500 RMB/month in ref. to 800 RMB/month
			1.93 (1.64 - 2.27)	< 0.001	1500-2500 RMB/month in ref. to 800 RMB/month
			2.88 (2.47 - 3.37)	< 0.001	> 2500 RMB/month in ref. to 800RMB/month
Phathammavong et al., 2008 [9]	536	OR	2.23 (1.04 - 4.81)	< 0.05	High income in ref. to low income: OR adjusted for gender, age, parity, parents education, pets ownership, sharing bed, air conditioning, measles infection, respiratory infection, time on road, meat, fish, vegetables, cow milk, fast food and eggs consumptions, and intestinal parasitic infestation
Parity					
Hsu et al., 2009 [10]	1368	OR	1.42 (1.02 - 1.97)	< 0.025	*N* = 1 in ref. to *N* ≥ 3 : OR adjusted for birth weight, gender, gestational age, maternal education, gestational complications, smoking, pets, carpets, molds, and air pollutions
			1.43 (1.01 - 2.01)	< 0.025	*N* = 2 in ref. to N ≥ 3: OR adjusted for birth weight, gender, gestational age, maternal education, gestational complications, smoking, pets, carpets, molds, and air pollutions
Family size					
Li et al.*,* 2015 [22]	20,803	OR	1.26 (1.05 - 1.51)	< 0.005	*N* < 3 in ref. to N ≥ 4
			1.18 (1.0 - 1.30)	< 0.005	*N* = 3 in ref. to *N* ≥ 4
Marital status					
An et al., 2015 [2]	31,217	OR	0.85 (0.74 - 0.97)	< 0.05	Married in ref. to unmarried: OR adjusted for age, gender, family size, residency, educational, Household income, and occupation

^a^*OR* odds ratio, *PR* prevalence ratio

**Fig. 1:**
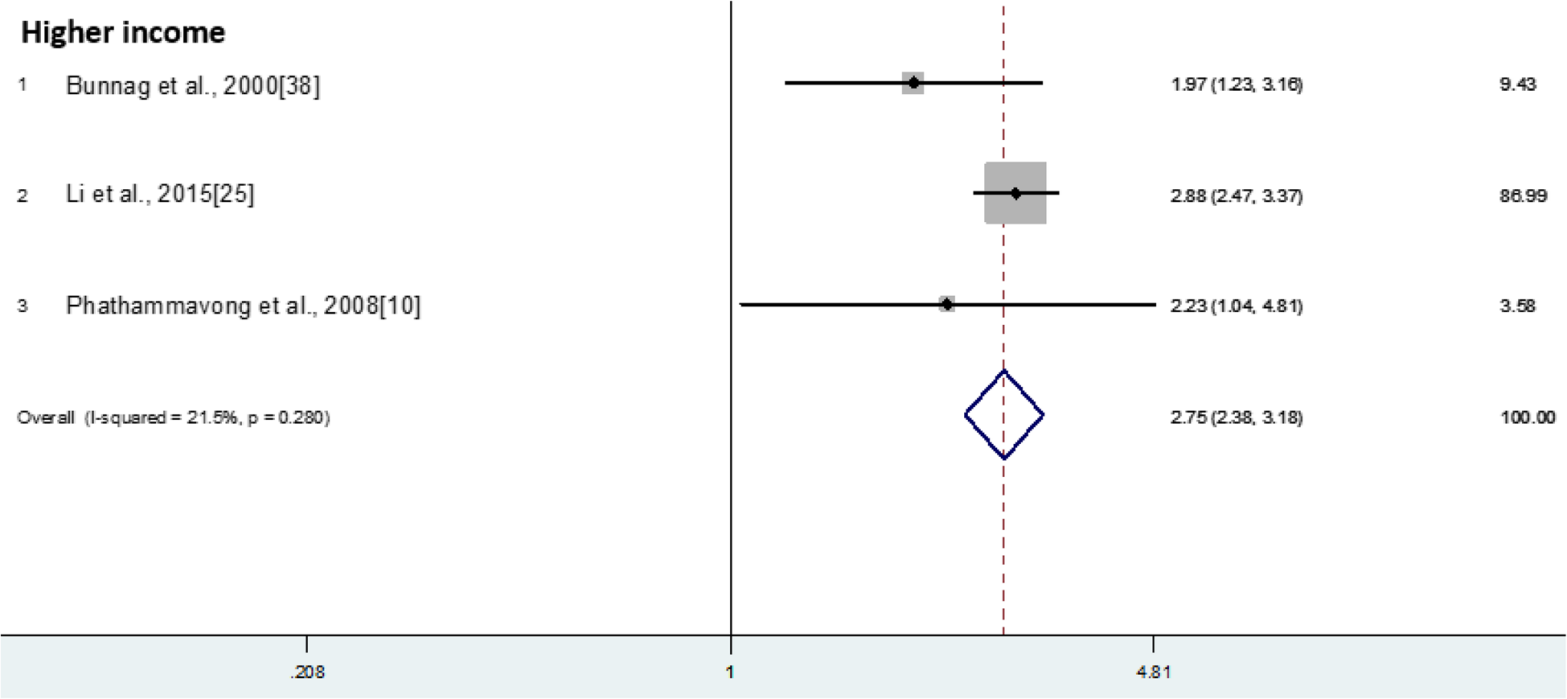
Individual and combined odds ratio and 95% confidence intervals for higher income group in association with Allergic Rhinitis presentation

In Li et al. [22], the odds of AR have shown to increase with the rise of household income when different household income groups are compared. For the household with an income of > 2500 RMB/month, the odds of AR is 2.88 times of those with an income of 800 RMB/month. A similar trend is observed in another two independent studies. A pooled odds ratio of 2.75 has been obtained which suggests the significant role of household income in affecting AR expression (Fig. 1).

Moreover, being married, a large number of members in the household, and parity were indicated to be beneficial for protecting one against AR. However, their influences towards protection of AR are likely to be interrelated as married individuals are usually with children and are therefore likely to report an increased parity number and household members.

### Personal risk factors affecting AR presentation

Apart from the demographical factors that are usually non-changeable to an individual, one’s behaviours, attitude, and encounters might have direct and indirect influences to the disease presentation. These factors are highly varied from one person to another and are often affected by their background and the social group they interact with.

**Fig. 2:**
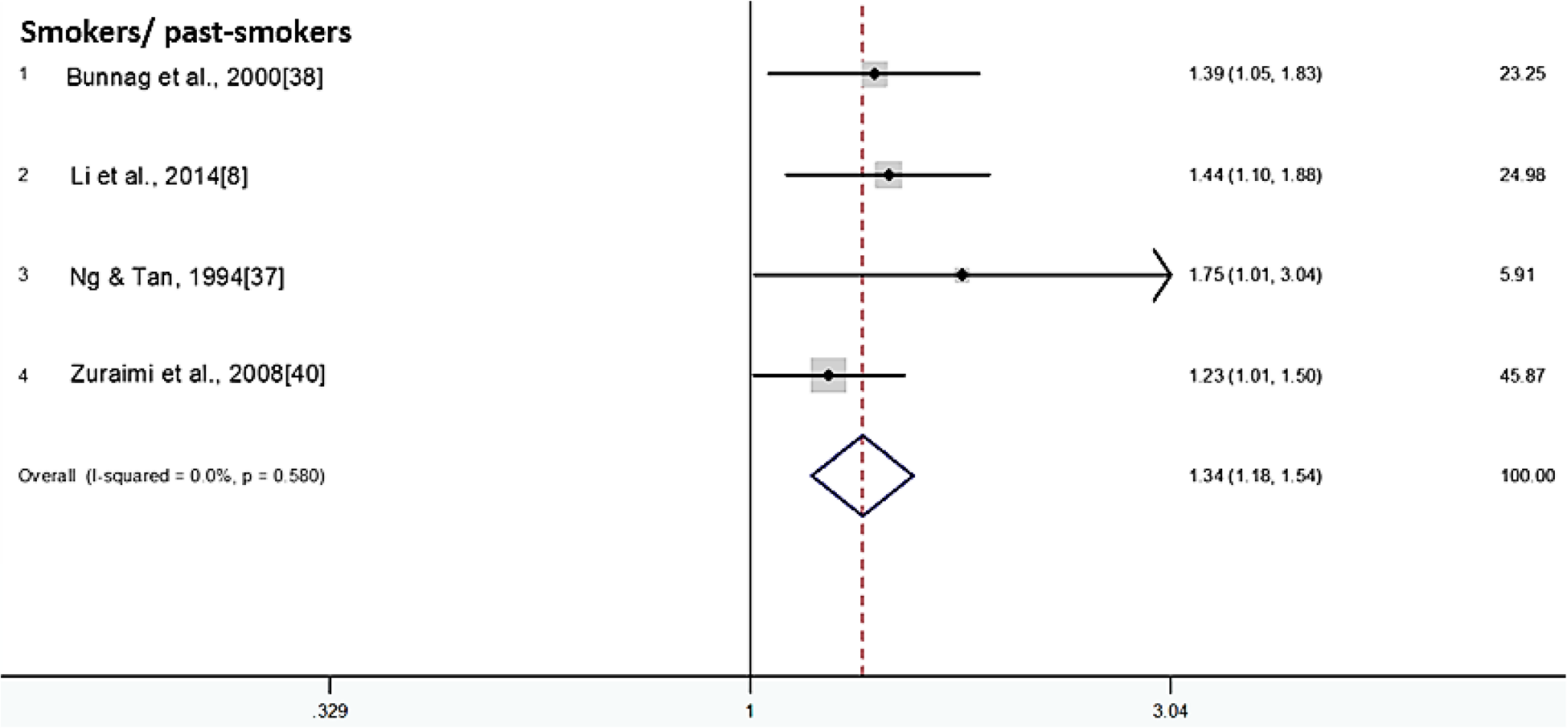
Individual and combined odds ratio and 95% confidence intervals for smokers/past-smokers in association with Allergic Rhinitis presentation

As stated in Table 5, like the case with many other infectious diseases [23, 24], alcohol consumption and smoking habits have shown to increase the odds of presenting AR. This is especially true for the smoking habit; which shows higher odds of expressing AR among present smokers, past smokers, and even passive smokers as compared to non-smokers and those who are not exposed to passive smoking. The result is consistent across four independent articles and a pooled odds ratio of 1.34 was obtained indicating smoking habit does associate with the increased AR manifestation (Fig. 2).

**Table 5:** Strength of association of personal risk factor with AR manifestation

Study	Study population, N	OR^a^	Values (95% CI)	*p*-value	References
Alcohol					
Bunnag et al., 2000 [37]	3124	OR	1.46 (1.15 - 1.86)	< 0.05	Drinker in ref. to non-drinker: adjusted OR
Smoking					
Bunnag et al., 2000 [37]	3124	OR	1.39 (1.05 - 1.83)	< 0.05	Smoker in ref. to non-smoker: adjusted OR
Li et al., 2014 [7]	9899	OR	1.44 (1.10 - 1.88)	< 0.01	Smoker in ref. to non-smoker
Ng & Tan, 1994 [36]	2868	OR	1.75 (1.01 – 3.04)	< 0.05	Past smoker in ref. to non- smoker: OR adjusted for age, flat size, housing estate, race, insect exposure, occupational exposure, and fume
Zuraimi et al., 2008 [39]	6794	OR	1.23 (1.01 - 1.50)	–	Passive smoker in ref. to non-passive smoker: OR adjusted for age, gender, race, socioeconomic status, housing type, family atopy, breastfeeding, food allergy, respiratory infections, home dampness, air conditioning, home wall paper, carpet, home traffic density, childcare centre ventilation and dampness
Computer usage					
Li et al., 2014 [7]	9899	OR	1.45 (1.10 - 1.91)	< 0.01	Occasionally in ref. to never
			1.46 (1.10 - 1.93)	< 0.01	< 2 h daily in ref. to never
			1.58 (1.14 - 2.19)	< 0.01	2-4 h daily in ref. to never
Education					
Alsowaidi et al., 2010 [3]	7550	OR	1.42 (1.05 - 1.93)	< 0.05	University in ref. to illiterate and primary school: OR adjusted for nationality, gender, family history of AR, and age
Min et al., 1997 [20]	10,054	OR	1.83 (0.82 - 4.02)	< 0.05	Elementary in ref. to illiterate: OR adjusted for age
			2.11 (0.93 - 4.79)	< 0.05	Junior in ref. to illiterate: OR adjusted for age
			2.81 (1.34 - 5.86)	< 0.05	Senior in ref. to illiterate: OR adjusted for age
			2.54 (1.08 - 5.96)	< 0.05	College in ref. to illiterate: OR adjusted for age
Stress					
An et al., 2015 [2]	31,217	OR	1.14 (1.01 - 1.28)	< 0.001	A little in ref. to little: OR adjusted for age, gender, height, weight, body mass index, smoking status, sleep time and drinking
			1.46 (1.28 - 1.66)	< 0.001	Moderate in ref. to little : OR adjusted for age, gender, height, weight, body mass index, smoking status, sleep time and drinking
			1.47 (1.21 - 1.79)	< 0.001	Severe in ref. to little
					: OR adjusted for age, gender, height, weight, body mass index, smoking status, sleep time and drinking
Sleep time					
An et al., 2015 [2]	31,217	OR	0.92 (0.84 - 1.00)	< 0.05	> 7 h in ref. to ≤7 h : OR adjusted for age, gender, height, weight, body mass index, smoking status, stress and drinking
Parasitic infection					
Phathammavong et al., 2008 [9]	536	OR	3.41 (1.03 - 11.29)	< 0.05	With parasitic infection in ref. to without : OR adjusted for gender, age, parity, parents education, pets ownership, sharing bed, air conditioning, measles infection, respiratory infection, time on road, meat, fish, vegetables, cow milk, fast food and eggs consumptions, and family income
Past respiratory infection					
Phathammavong et al., 2008 [9]	536	OR	4.06 (1.83 - 9.01)	< 0.05	With past respiratory infection in ref. to without : OR adjusted for gender, age, parity, parents education, pets ownership, sharing bed, air conditioning, measles infection, family income, time on road, meat, fish, vegetables, cow milk, fast food and eggs consumptions, and intestinal parasitic infestation

^a^*OR* odds ratio

Coincidentally, people with more computer usage, higher education, higher stress level and lesser sleeping time were presented with higher AR susceptibility. Though several pathways were speculated for such association, the effects of confounders and bias could not be ruled out and further study is required to establish the direct association link between these factors.

Stress might be one of the critical risk factors for AR presentation. Studies have shown the association between the level of stress in individuals with more frequent drinking and smoking habits, having higher daily computer usage, and higher education levels but with less sleeping time [25–27]. Being in a stressful situation can trigger the expression of cortisol which can induce allergic responses and enhance AR expression (Table 8) [28]. In addition, literature has suggested the possibility of dust trapped on the computer [7] and higher indoor allergen exposure [2] to explain the higher odds of AR manifestation among office workers who usually have higher education qualifications. Dose-response effects were also observed in computer usage, education attainment and stress level as odds of AR increase with higher level of risk exposure, with the exception for AR odds of college students to illiterate individuals in Min et al. [20].

In contrast, people with parasitic or past respiratory infections were reported to have higher odds of AR presentation. The results are contradictory with biological plausibility discussed in other literature. Phathammavong et al. [9], proposed that AR and other respiratory infections compete for immune responses, resulting in a higher odds of presenting AR among the respiratory infection patients. This hypothesis is supported by the reported odds of AR for individuals with either parasitic infection or past respiratory infection are exceptionally high (3.41 and 4.06 respectively). However, this factor has only been studied in Phathmmavong et al. among the articles reviewed and further analysis is essential to confirm the effects of these infections on AR presentation, which could be one of the most important factors in predicting AR risk.

### Family risk factors affecting AR presentation

In Table 6, mother depression and cesarean delivery are positively correlated with the odds of AR presentation. As stated in Li et al. [22], pre- and postnatal depression stimulates the production of cortisol, and this secretion affects the immune development of a fetus and increases the odds of presenting AR. Apart from this, cesarean delivery might further exacerbate this situation as unlike vaginal delivery, the infants are not exposed to the mother’s birth canal microflora, which has shown to be protective against AR expression [29] as illustrated in Table 8.

**Table 6:** Strength of association of family factor with AR manifestation

Study	Study population, N	OR/PR^a^	Values (95% CI)	*p*-value	References
Age of gestation					
Lee et al., 2017 [42]	1497	OR	0.51	< 0.05	Preterm in ref. to term
Li et al., 2015 [22]	20,803	OR	1.07 (0.88 - 1.30)	< 0.001	Preterm in ref. to term
			1.42 (1.20 - 1.69)	< 0.001	Post-term in ref. to term
Mother depression					
Li et al., 2015 [22]	20,803	OR	1.16 (1.05 - 1.29)	< 0.05	Mother with pre- or postnatal depression in ref. to without
Mode of delivery					
Li et al., 2015 [22]	20,803	OR	1.36 (1.23 - 1.49)	< 0.001	Cesarean in ref. to vaginal delivery
Breastfeeding					
Huang et al., 2017 [34]	13,335	OR	0.97 (0.94 - 0.99)	< 0.05	With exclusive for > 6 months breastfeeding in ref. to never breastfeeding : OR adjusted for family atopy, gender, age, district of the current residence, home ownership, early pet-keeping, parental smoking, and home dampness
Li et al., 2015 [22]	20,803	OR	0.67 (0.61 – 0.73)	< 0.001	With exclusive breastfeeding in the first 4 months in ref. to without
Maternal education					
Li et al., 2015 [22]	20,803	OR	1.55 (1.36 - 1.77)	< 0.001	High school in ref. to middle school or below
			2.11 (1.86 - 2.39)	< 0.001	College or above in ref. to middle school or below
Paternal education					
Li et al., 2015 [22]	20,803	OR	1.52 (1.32 - 1.74)	< 0.001	High school in ref. to middle school or below
			2.02 (1.77 - 2.30)	< 0.001	College or above in ref. to middle school or below
Gruel introduction					
Huang et al., 2017 [34]	13,335	OR	0.95 (0.90 - 1.00)	< 0.05	For > 6 months-old in ref. to < 3 months-old : OR adjusted for family atopy, gender, age, district of the current residence, home ownership, early pet-keeping, parental smoking, and home dampness
Family history of atopy					
Bunnag et al., 2000 [37]	3124	OR	1.96 (1.56 - 2.46)	< 0.05	With family history of atopy in ref. to without: adjusted OR
Family history of allergic diseases					
Alsowaidi et al., 2010 [3]	7550	OR	6.08 (4.93 - 7.50)	< 0.0005	With family history of AR in ref. to without : OR adjusted for nationality, gender, age, and education
Li et al., 2014 [7]	9899	OR	3.51 (2.65 - 4.64)	< 0.001	With family history of AR in ref. to without
Graif et al., 2004 [38]	10,057	OR	1.30 (1.02 - 1.66)	–	With family history of asthma in ref. to without: OR adjusted for current asthma, gender, gender, gender, and smoking
Norbäck et al., 2016 (2) [35]	462	OR	3.49 (1.97 - 6.20)	< 0.001	With family history of allergic reactions in ref. to without: OR adjusted for gender, smoking, atopy, and race
Ziyab, 2017 [8]	1154	PR	1.82 (1.39 - 2.39)	< 0.001	With maternal allergy in ref. to without: PR adjusted for gender, cat exposure, and age
			1.87 (1.25 - 2.77)	< 0.005	With paternal allergy in ref. to without: PR adjusted for gender, cat exposure, and age

^a^*OR* odds ratio, *PR* prevalence ratio

Conversely, inconsistent results are observed for the association of breastfeeding with AR presentation across multiple studies [30, 31]. This refutes the commonly accepted hypothesis which states breastfeeding as protective through the antibodies present in the milk and the additional nutrients from the mother’s diet transferred to the milk [32, 33]. In contrast, parental education and awareness encourages a hygienic environment which is unfavorable for AR protection as this reduces the chance of exposing their children to a larger variety of allergens in early life. Similarly, for gruel consumption, the subtle protection might be due to the effect of gruel to stimulate inflammatory cytokines which suppress the allergic reaction [34].

On the other hand, genetic factor is long established to play an influential role in AR presentation [15] and a family history of atopy and allergic diseases might predispose children to AR. Multiple studies have shown that family history is a key risk factor associated with the increased risk of AR expression. This is particularly true for children with a family history of AR as high odds ratios of 6.08 and 3.51 have been reported in studies conducted by Alsowaidi et al. [3] and Li et al. [7], respectively. However, genetic factor is non-modifiable and hence, it needs to be complemented with other preventive measures in order to reduce the risk of presenting the disease.

### Environmental risk factors affecting AR presentation

As suggested in multiple studies investigated, environmental factors are highly important in triggering AR. For instance, Table 7 has shown that the presence of allergens such as fungi, molds, insects and house dust mites could increase the odds of presenting AR. Among the allergens studied, the presence of fungi and molds were reported to have very high odds of association to AR with 3.44 for fungi in Norbäck et al. [35] and 9.40 for molds in Kidoni et al. [19] Moreover, insect exposure and house dust mite have been identified as two of the most important risk factors for AR as indicated in Table 7. These common indoor allergens, such as mold and fungal spores, insect wastes and house dust mite fecal proteins can induce Type I hypersensitivity reaction by promoting the expression of a range of allergic-causing mediators, thus increasing the odds of expressing AR (Table 8). In addition, the utilization of carpets, which trap dust, and home renovation, which introduces a variety of allergic-causing renovation materials, further exacerbate the situation.

**Table 7:** Strength of association of environmental risk factors with AR manifestation

Study	Study population, N	OR/PR^a^	Values (95% CI)	*p*-value	References
Fungi					
Norbäck et al.*,* 2016 (1) [41]	462	OR	0.76 (0.58 - 0.99)	< 0.05	With fungi in ref. to without: OR adjusted for gender, ethnicity, smoking, atopy and heredity
Norbäck et al.*,* 2016 (2) [35]	462	OR	3.44 (1.81 - 6.59)	< 0.001	With fungal endotoxin C14 3-OH FA in ref. to without: OR adjusted for classroom level
Mold					
Kidoni et al., 2004 [19]	202	OR	9.40 (3.80 - 22.90)	–	With mold sensitization vs without
Insect					
Ng & Tan, 1994 [36]	2868	OR	2.08 (1.29 – 3.35)	< 0.005	Once every day in ref. to once every few months : OR adjusted for age, flat size, housing estate, race, race, occupational exposure, and fume
House dust mite					
Lim et al.*,* 2015 [11]	695	OR	1.66 (1.08 - 2.56)	< 0.05	With house dust mite allergy in ref. to without : OR adjusted for gender, current smoking status, age, cat allergy, home dampness, and indoor home painting in last 12 months
Norbäck et al.*,* 2016 (2) [35]	462	OR	2.91 (1.35 - 6.24)	< 0.01	Continuous variable, 1000 mg increase in fine dust : OR adjusted for classroom level
Carpet					
Hsu et al., 2009 [35]	1368	OR	1.60 (1.09 - 2.35)	< 0.025	With carpets in ref. to without : OR adjusted for birth weight, gender, gestational age, maternal education, gestational complications, smoking, pets, parity, molds, and air pollutions
Home renovation					
Li et al.*,* 2014 [7]	9899	OR	1.39 (1.06 - 1.81)	< 0.05	With home renovation in ref. to without
Air pollution					
Hsu et al.*,* 2009 [10]	1368	OR	1.44 (1.10 - 1.88)	< 0.01	With air pollution in ref. to without : OR adjusted for birth weight, gender, gestational age, maternal education, gestational complications, smoking, pets, carpets, molds, and parity
Fume exposure					
Ng & Tan, 1994 [36]	2868	OR	2.29 (1.32 - 3.99)	< 0.005	Often in ref. to rarely: OR adjusted for age, flat size, housing estate, race, race, occupational exposure, and race
Traffic					
Zuraimi et al.*,* 2011 [21]	2994	PR	1.58 (1.04 - 2.39)	< 0.05	Heavy traffic in ref. to low traffic for all children : PR adjusted for gender, age, race, socioeconomic status, housing type, parental atopy, breastfeeding, food allergy, and resident height
			1.73 (1.00 - 2.99)	< 0.05	Heavy traffic in ref. to low traffic for all lifetime residents : PR adjusted for gender, age, race, socioeconomic status, housing type, parental atopy, breastfeeding, food allergy, and resident height
Occupational exposure					
An et al.*,* 2015 [2]	31,217	OR	1.28 (1.11 - 1.47)	< 0.01	Unemployed in ref. to engineer : OR adjusted for age, gender, family size, residency, educational, household income, and marriage
			1.29 (1.09 - 1.52)	< 0.01	Manager, expert, specialist & clerks in ref. to engineer : OR adjusted for age, gender, family size, residency, educational, household income, and marriage
			1.18 (1.01 - 1.39)	< 0.01	Service worker & seller in ref. to engineer : OR adjusted for age, gender, family size residency, educational, household income, and marriage
			1.32 (1.11 - 1.58)	< 0.01	Technician, mechanics & production worker in ref. to engineer : OR adjusted for age, gender, family size, residency, educational, household income, and marriage
Ng & Tan 1994 [36]	2868	OR	1.95 (1.36 - 2.80)	< 0.0005	Wth occupational exposure vs without: OR adjusted for age, flat size, housing estate, race, race, fume, and race

^a^*OR* odds ratio, *PR* prevalence ratio

**Table 8:** Collated potential risk factors for AR presentation

No	Potential risk factor	No. of studies	No. of studies with significant results	Possible explanations	Sources
1	Age	7	5	The allergic condition is highest in young adults, declining with age [43]. However, the reason remains unclear.	Alsowaidi et al.*,* 2010 [3] Li et al.*,* 2015 [22] Lim et al., 2015 [11] Ng & Tan, 1994 [36] Ziyab, 2017 [8] Li et al.*,* 2014^a^ [7] Phathammavong et al., 2008^a^ [9]
2	Age of gestation	3	2	Preterm baby, who is characterized by lower birth weight and earlier exposure to the mother microflora, have prematurity protection against AR [42]. In contrast, successful pregnancy shifted the T lymphocytes production to Th2 which increases the risk of atopy and AR [22].	Lee et al.*,* 2017 [42] Li et al.*,* 2015 [22] Hsu et al., 2009^a^ [10]
3	Air conditioning	2	0	Home dampness has been shown to be related to allergic rhinitis exacerbations [44], probably in relation to the development of mold or mildew. As air-conditional areas usually have higher dampness, it may lead to increase in AR expression [45].	Phathammavong et al., 2008^a^ [9] Zuraimi et al., 2011^a^ [21]
4	Air pollution	2	1	The pollutants might provoke and exacerbate the allergic conditions of the current patients. Besides, it might also make a person more susceptible to certain allergens [45].	Hsu et al., 2009 [10] Ng & Tan, 1994^a^ [36]
5	Alcohol consumption (self/parent)	3	1	Alcohol consumption is related to increased stress level which is one of the provoking factors potentially enhancing AR presentation [2].	Bunnag C et al., 2000 [37] An et al., 2015^a^ [2] Li et al., 2015^a^ [22]
6	Birth order	2	0	An allergic mother might be more prone to provide low-exposure environment for the next children [29].	Phathammavong et al., 2008^a^ [9] Ziyab, 2017^a^ [8]
7	BMI	2	0	Higher BMI and greater weight-to-height ratio is associated with higher atopic and higher allergic diseases incidence regardless of gender and age [43].	An et al.*,* 2015^a^ [2] Lei, Yang & Zhen, 2016^a^ [40]
8	Breastfeeding	3	2	Breastfeeding for more than 6 months has shown to enhance the presentation of AR [30, 31], but the reason remains unknown. Contrary plausibility has also shown that food proteins consumed by the mother [32] or breastfeeding might help to reduce the inflammatory responses by destroying microbes [33] and is thus protective against AR presentation.	Huang et al.*,* 2017 [34] Li et al., 2015 [22] Li et al., 2014^a^ [7]
9	Car ownership	1	0	Car owners spend more time outdoor and are thus exposed to higher levels of outdoor pollutants [46].	Li et al., 2014^a^ [7]
10	Carpet	2	1	Having carpets at home increases the risk of accumulating mite allergens, thus resulting in more AR cases [47].	Hsu et al., 2009 [10] Ng & Tan, 1994^a^ [36]
11	Computer usage	1	1	Studies suggested that when the computer is not properly cleaned, prolong usage of the computer will likely result in higher allergen exposure and thus an increase in AR cases [7].	Li et al., 2014 [7]
12	Drinking (self/parent)	3	1	Alcohol consumption is related to increased stress level which is one of the provoking factors potentially enhancing AR presentation [2].	Bunnag C et al., 2000 [37] An et al., 2015^a^ [2] Li et al., 2015^a^ [22]
13	Education attainment	3	2	People with higher education usually work in an indoor environment, thus exposing them to indoor allergens [2].	Alsowaidi et al., 2010 [3] Min et al., 1997 [20] An et al.*,* 2015^a^ [2]
14	Family history of allergic diseases	6	5	Allergic diseases can be hereditary, with incomplete genetic penetrance [48].	Alsowaidi et al., 2010 [3] Li et al.*,* 2014 [7] Graif et al.*,* 2004 [38] Norbäck et al., 2016 (2) [35] Ziyab, 2017 [8] Phathammavong et al., 2008^a^ [9]
15	Family history of atopy	1	1	Atopy is usually used as a marker for other allergic diseases, and genetic factors usually play a role in allergic disease presentation. As such, higher family history of atopy usually suggests higher chance of contracting allergic diseases [43].	Bunnag et al., 2000 [37]
16	Family size	2	1	Crowding increases the contact of an individual with allergens and is thus protective against manifestation of allergic reaction [47].	Li et al.*,* 2015 [22] An et al.*,* 2015^a^ [2]
17	Food	1	0	Some foods are protective against AR, most likely through shifting the macromolecules production, such as fatty acid balance, which later results in the reduction of inflammatory mediators required for disease presentation [30].	Phathammavong et al., 2008^a^ [9]
18	Fume exposure	1	1	Fume released into the air by various means is also one of the potential triggering factors in AR presentation [45].	Ng & Tan, 1994 [36]
19	Fungi	2	2	Airborne fungi spores induce type I hypersensitivity and hence AR presentation [49].	Norbäck et al.*,* 2016 (1) [41] Norbäck et al.*,* 2016 (2) [35]
20	Gender	12	7	The allergic diseases appear more frequently in males at infant age, but with equal burden as females at mid-teens, and then become more frequent in females with the reason remain largely unknown [43].	Alsowaidi et al., 2010 [3] Graif et al., 2004 [38] Hsu et al.*,* 2009 [10] Lee et al.*,* 2017 [42] Lei, Yang & Zhen, 2016 [40] Li et al., 2015 [22] Bunnag et al.*,* 2000^a^ [37] Lim et al., 2015^a^ [11] Ng & Tan, 1994^a^ [36] Norbäck et al., 2016 (2)^a^ [35] Phathammavong et al.*,* 2008^a^ [9] Ziyab, 2017^a^ [8]
21	Gestational complication	1	0	Uterus complication during gestation periods affects the immune system development of the fetus and increases the risk of atopy-related diseases [29].	Hsu et al.*,* 2009^a^ [10]
22	Gruel introduction period	1	1	Study shows that gruel introduction between 4 to 6 months, in complementary with breastfeeding, induces IL-10 and TGFβ production which is protective against AR [34].	Huang et al.*,* 2017 [34]
23	Hair coloring	1	0	Oxidative hair dye can induce hypersensitivity reactions, thus increasing the risk of expressing AR [50].	Li et al., 2014^a^ [7]
24	Home renovation	1	1	The materials used during the home renovation, such as formaldehyde might have an impact in causing cell sensitization and later AR presentation [7, 31].	Li et al.*,* 2014 [7]
25	House dust mite	2	2	Long term exposure to threshold concentrations of dust mite fecal proteins causes the presentation of allergens by antigen presenting cells (APC) to CD4+ T lymphocytes, leading to the production of downstream mediators and manifestation of AR symptoms [49].	Lim et al., 2015 [11] Norbäck et al.*,* 2016 (2) [35]
26	Household income	5	3	Higher income is associated with better living conditions and hygiene behavior, thus reducing the exposure to a variety of allergens, which possibly increases their odds of AR [42].	Bunnag et al., 2000 [37] Li et al., 2015 [22] Phathammavong et al., 2008 [9] An et al., 2015^a^ [2] Li et al., 2014^a^ [7]
27	Housing estate	2	2	Living in a housing estate with poor environmental conditions has resulted in more allergic cases [47].	Li et al., 2015 [22] Ng & Tan, 1994 [36]
28	Insect	1	1	Prolonged exposure to insects, which is one of the common allergens may trigger hypersensitivity reactions with production of mediators and hence, the expression of AR symptoms [49].	Ng & Tan, 1994 [36]
29	Marital status	2	1	Being married is hypothesized to be associated with positive physical and mental outcomes and is therefore protective against AR [2].	An et al., 2015 [2] Min et al.*,* 1997^a^ [20]
30	Maternal education	2	1	Educated parents will have higher awareness of their children health status, and thus adopt protective measures to combat against AR starting from a young age [45].	Li et al., 2015 [22] Hsu et al.*,* 2009^a^ [10]
31	Maternal pre- or postnatal depression	1	1	Pre- or postnatal depression results in excessive cortisol expression, which will affect the immune system development of the fetus [22].	Li et al., 2015 [22]
32	Mode of delivery	2	1	Exposure of the fetus to the mother microflora during birth is an advantage to protect them against allergic sensitization [29, 51]. In contrast, cesarean birth is associated with higher AR risk [51].	Li et al., 2015 [22] Ziyab, 2017^a^ [8]
33	Mold	2	1	Mold spores induce type I hypersensitivity and hence, AR presentation [49].	Kidoni et al., 2004 [19] Hsu et al.*,* 2009^a^ [10]
34	Nationality	1	1	AR prevalence is especially high in Asia probably due to the higher humidity, more extensive smoking and vaccination habits [43].	Alsowaidi et al., 2010 [3]
35	Occupational exposure	3	2	Some occupations have higher risk of exposure to allergens, thus increasing their risk of expressing AR [50].	An et al., 2015 [2] Ng & Tan, 1994 [36] Min et al.*,* 1997^a^ [20]
36	Parasitic infection	1	1	Parasitic infection might have some effects to a person’s gut microbiota, which could later offer some protection against allergic sensitization as stated in hygiene hypothesis [52]. However, some literature also show that parasitic infection influences the allergy development due to its competition with human immune response [9].	Phathammavong et al., 2008 [9]
37	Parity	2	1	Being allergic might cause reduced reproductivity in females, resulting in a lower parity which is associated with AR presentation [29].	Hsu et al.*,* 2009 [10] Phathammavong et al., 2008^a^ [9]
38	Past measles infection	1	0	The association of measles with AR is not clear, but it was hypothesized that measles infection might protect against AR development or could promote allergic sensitization [52].	Phathammavong et al.*,* 2008^a^ [9]
39	Past respiratory infection	1	1	Evidence shows that past respiratory infection, such as tuberculosis caused by *Mycobacterium tuberculosis* could be protective against AR, possibly through reduction of allergy sensitization [52]. In contrast, some studies have shown that past respiratory infection is directly associated with AR development [9].	Phathammavong et al., 2008 [9]
40	Paternal education	1	1	Educated parents are more likely to keep a hygienic living environment, thus possibly increasing the incidence of allergic conditions in their children [45].	Li et al.*,* 2015 [22]
41	Pet	6	3	For individuals sensitive to pet furs, long term exposure to the pet induces hypersensitivity reaction and could later result in AR presentation [49].	Li et al.*,* 2014 [7] Ziyab, 2017 [8] Phathammavong et al., 2008^a^ [9] Hsu et al.*,* 2009^a^ [10] Lim et al., 2015^a^ [11] Ng & Tan, 1994^a^ [36]
42	Race	3	3	Cultural differences between the races probably have some effects on AR presentation; however, there is currently no specific research addressing the impact of races on AR disease presentation.	Graif et al.*,* 2004 [38] Ng & Tan, 1994 [36] Norbäck et al.*,* 2016 (2) [35]
43	Residency	5	4	For people who lived in urban areas, they are more prevalent in developing allergic reaction [47], probably due to a poorer housing or environmental conditions. Modern building techniques increase indoor humidity and temperature, facilitates mold development and hence, contributes to AR presentation [2].	Graif et al.*,* 2004 [38] Lee et al., 2017 [42] Li et al.*,* 2014 [7] Min et al.*,* 1997 [20] An et al.*,* 2015^a^ [2]
44	Sharing bed	1	0	Sharing bed is hypothesized as one of the potential risk factors for AR [9], probably due to increased risk of getting infections from other people.	Phathammavong et al.*,* 2008^a^ [9]
45	Sleep time	1	1	People with lesser sleep are usually with higher levels of stress, which is a potential trigger factor for AR expression [2].	An et al., 2015 [2]
46	Smoking (self/parent)	12	4	Tobacco smoke is one of the trigger factors which precipitates the hypersensitivity reactions, thus exacerbating the AR conditions [47]. On the other hand, parents with AR children will also try to reduce their children exposure to external allergic stimuli through changing their smoking habits, thus explaining the negative association of AR and smoking habit [45].	Bunnag et al., 2000 [37] Li et al.*,* 2014 [7] Ng & Tan, 1994 [36] Zuraimi et al.*,* 2008 [39] An et al., 2015^a^ [2] Hsu et al.*,* 2009^a^ [10] Li et al.*,* 2015^a^ [22] Lim et al.*,* 2015^a^ [11] Min et al., 1997^a^ [20] Norbäck et al.*,* 2016 (2)^a^ [35] Phathammavong et al.*,* 2008^a^ [9] Ziyab, 2017^a^ [8]
47	Social class	1	0	As stated in hygiene hypothesis, people in lower social class are likely to have a greater exposure to infections. This may have direct and indirect impacts to their gut microbiota, which might offer protection against allergic sensitization [45, 52].	Min et al.*,* 1997^a^ [20]
48	Stress level	1	1	Stress can trigger the production of cortisol, and later induce allergic responses [28].	An et al., 2015 [2]
49	Time on road	1	0	Longer time spent on road is associated with higher AR risk, probably due to prolonged exposure to air contaminant [9].	Phathammavong et al.*,* 2008^a^ [9]
50	Traffic	1	1	The release of motor vehicles such as NO_x_ and CO provokes and exacerbates the conditions of the current AR patients, and might have consequences on changes in susceptibility towards allergens, thus affecting AR presentation [45]. Depending on the outdoor environmental pollution, long term exposure to heavy traffic might lead to allergic sensitization and resulted in AR expression [21].	Zuraimi et al., 2011 [21]

^a^Indicates the publication with insignificant results

Similarly, outdoor exposures to heavy traffic, air pollution, and fume exposure were also reported to be positively correlated with AR manifestation. These factors are especially crucial for those whose occupations expose them to the allergens [36]. Constant outdoor encounters with pollutants released from motor vehicles and heavy fumes during work promote AR presentation by changing a person’s susceptibility towards allergens [2, 10, 21, 36].

### Evaluation of risk factors associated with AR manifestation using several criteria

Various risk factors have shown strong association with AR presentation. Results are consistent for several risk factors across studies with different experimental setups and countries.

In addition to the ORs, criteria such as biological gradient, biological plausibility and temporality are important in evaluating the association between risk factors and AR. The biological gradient of the factor can be established especially when it is studied in a continuous manner or in multiple exposure levels. This was demonstrated in various demographical factors such as in family income, family size, personal factors like computer usage, education attainment, stress levels and even in parental education attainment. Moreover, the association between the risk factors and AR manifestation are further strengthened when factors with similar roles in AR presentation, such as the common allergens like house dust mites, fungi, and molds, display comparable results.

Furthermore, the listed factors can only be considered as a potential risk if its exposure is reasonably affected or altered the risk of AR development. Its biologic plausibility must also be coherent to the study results found. However, with reference to Table 7, breastfeeding, parasitic infection and past respiratory infections show contradictory results as to what is hypothesized and further analysis and interpretation is thus needed.

**Fig. 3:**
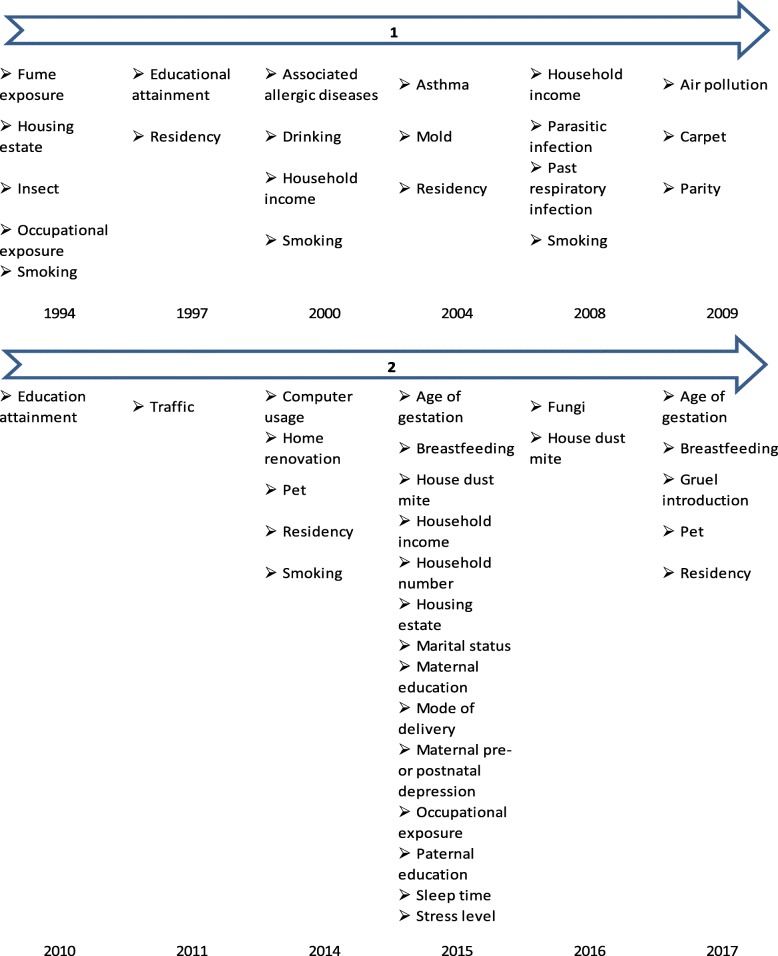
Years in which significant AR risk factors were identified

Last but not least, with reference to Fig. 3, the two risk factors, family income and smoking, analyzed using meta-analysis are consistently being identified as significant AR risk factor before and after 2010. In addition, education attainment and occupational exposure are two other significant modifiable risks that appeared in AR publications before and after 2010 in Asia. In contrast, it was observed that after the year 2010, more family-related risks were analyzed and shown to be significant AR risk factors, such as the age of gestation and breastfeeding. This suggests a shift in focus to consider more family-related risk factors among the Asian population.

### Limitations and conclusion

The studies chosen for this review are limited to articles published in Asia. Thus, the result might not be relevant and applicable to other nations outside Asia. In addition, the analysis might still be biased though several criteria have been used in establishing the significance of the potential AR risk factor in triggering or protecting against AR presentation. The analysed data could be affected by personal viewpoints in addition to errors occurred when translating data from primary literature to the review summaries, such as misrepresentation and misinterpretation of the original data. Thus, it is highly recommended for readers to refer to the original articles before extracting any information from this article. Furthermore, as most of the studies used in this review are observational studies, confounding effects cannot be ruled out and the association of a particular risk factor with the disease presentation might not be as straightforward as what is illustrated here.

From the articles reviewed, family income, family size, computer usage, personal and parental education attainment and stress level are identified as risk factors with the greatest potential to influence AR presentation, and when compared to other factors, they fulfill most of the criteria listed. In contrast, more considerations are required in interpreting the effects of breastfeeding, parasitic infections and past respiratory infections to AR presentation. These factors show incoherent biological plausibility and more in-depth investigation and analysis is thus required.

The results obtained from this review article can be used to improve the diagnosis of AR in clinical settings by identifying patients with risk factors strongly associated with AR manifestation. In addition, as personal and family-related modifiable factors are found to be strong AR triggering factors, strategies to alleviate personal stress levels and increase the awareness of allergy risk in a hygienic environment have to be developed.

## Additional file

Additional file 1: List of countries and dependent territories used in the literature review search. (PDF 322 kb)

## Abbreviations

AR: Allergic rhinitis
OR: Odds ratio
PR: Prevalence ratio

## References

[1] Strachan D, Sibbald B, Weiland S, Aït-Khaled N, Anabwani G, Anderson HR (1997). Worldwide variations in prevalence of symptoms of allergic rhinoconjunctivitis in children: the international study of asthma and allergies in childhood (ISAAC). Pediatr Allergy Immunol.

[2] An S-Y. Analysis of various risk factors predisposing subjects to allergic rhinitis. Asian Pacific J Allergy Immunol. 2015;:143–52. doi:10.12932/AP0554.33.2.2015. 10.12932/AP0554.33.2.2015 26141036

[3] Alsowaidi S, Abdulle A, Shehab A, Zuberbier T, Bernsen R (2010). Allergic rhinitis: prevalence and possible risk factors in a gulf Arab population. Allergy Eur J Allergy Clin Immunol..

[4] Bousquet J (2008). Allergic rhinitis and its impact on asthma (ARIA) 2008. Allergy.

[5] Murray CS (2004). Allergens, viruses, and asthma exacerbations. Proc Am Thorac Soc.

[6] Cookson W. The alliance of genes and environment in asthma and allergy. Nature 1999;402 November:B5–11. 10.1038/35037002 10586889

[7] Li CW, De Chen H, Zhong JT, Bin LZ, Peng H, Lu HG (2014). Epidemiological characterization and risk factors of allergic rhinitis in the general population in Guangzhou City in China. PLoS One.

[8] Ziyab AH (2017). Prevalence and risk factors of asthma, rhinitis, and eczema and their multimorbidity among young adults in Kuwait: a cross-sectional study. Biomed Res Int.

[9] Phathammavong O, Ali M, Phengsavanh A, Xaysomphou D, Odajima H, Nishima S, et al. Prevalence and potential risk factors of rhinitis and atopic eczema among schoolchildren in Vientiane capital, Lao PDR: ISAAC questionnaire. Biosci Trends 2008;2:193–199. 20103927

[10] Hsu S-P, Lin K-N, Tan C-T, Lee F-P, Huang H-M (2009). Prenatal risk factors and occurrence of allergic rhinitis among elementary school children in an urban city. Int J Pediatr Otorhinolaryngol.

[11] Lim FL, Hashim Z, LTL T, Said SM, Hashim JH, Norbäck D (2015). Asthma, airway symptoms and rhinitis in office workers in Malaysia: associations with house dust mite (HDM) allergy, cat allergy and levels of house dust mite allergens in office dust. PLoS One.

[12] Tamay Z, Akcay A, Ones U, Guler N, Kilic G, Zencir M (2007). Prevalence and risk factors for allergic rhinitis in primary school children. Int J Pediatr Otorhinolaryngol.

[13] Sultész M, Katona G, Hirschberg A, Gálffy G (2010). Prevalence and risk factors for allergic rhinitis in primary schoolchildren in Budapest. Int J Pediatr Otorhinolaryngol.

[14] Kuyucu S, Saraclar Y, Tuncer A, Geyik PO, Adalioglu G, Akpinarli A (2006). Epidemiologic characteristics of rhinitis in Turkish children: the international study of asthma and allergies in childhood (ISAAC) phase 2. Pediatr Allergy Immunol.

[15] Magnan A, Meunier JP, Saugnac C, Gasteau J, Neukirch F (2008). Frequency and impact of allergic rhinitis in asthma patients in everyday general medical practice: a French observational cross-sectional study. Allergy Eur J Allergy Clin Immunol.

[16] Cirillo I, Marseglia G, Klersy C, Ciprandi G (2007). Allergic patients have more numerous and prolonged respiratory infections than nonallergic subjects. Allergy Eur J Allergy Clin Immunol..

[17] Cardell LO, Olsson P, Andersson M, Welin KO, Svensson J, Tennvall GR, et al. TOTALL: high cost of allergic rhinitis - a national Swedish population-based questionnaire study. npj Prim Care Respir Med 2016;26. 10.1038/npjpcrm.2015.82 PMC4741287 26845513

[18] Weiss KB, Sullivan SD (2001). The health economics of asthma and rhinitis. I. Assess Econ Impact. J Allergy Clin Immunol.

[19] Kidoni MI, See Y, Goh A, Chay OM, Balakrishnan A (2004). Aeroallergen sensitization in pediatric allergic rhinitis in Singapore: is air-conditioning a factor in the tropics?. Pediatr Allergy Immunol.

[20] Min YG, Jung HW, Kim HS, Park SK, Yoo KY. Prevalence and risk factors for perennial allergic rhinitis in Korea: results of a nationwide survey. Clin Otolaryngol Allied Sci. 1997;22:139–44. 10.1046/j.1365-2273.1997.00879.x 9160927

[21] Zuraimi MS, Tham KW, Chew FT, Ooi PL, Koh D (2011). Home air-conditioning, traffic exposure, and asthma and allergic symptoms among preschool children. Pediatr Allergy Immunol.

[22] Li Y, Jiang Y, Li S, Shen X, Liu J, Jiang F (2015). Pre-and postnatal risk factors in relation to allergic rhinitis in school-aged children in China. PLoS One.

[23] Talamini G, Bassi C, Falconi M, Sartori N, Salvia R, Rigo L (1999). Alcohol and smoking as risk factors in chronic pancreatitis and pancreatic cancer. Dig Dis Sci.

[24] Poikolainen K, Karvonen J, Pukkala E (1999). Excess mortality related to alcohol and smoking among hospital-treated patients with psoriasis. Arch Dermatol.

[25] Conway TL, Vickers RR, Ward HW, Rahe RH (1981). Occupational stress and variation in cigarette, coffee, and alcohol consumption. J Health Soc Behav.

[26] Robotham D, Julian C (2006). Stress and the higher education student: a critical review of the literature. J Furth High Educ.

[27] Jacobsen LK, Southwick SM, Kosten TR (2001). Substance use disorders in patients with posttraumatic stress disorder : a review of the literature. Am J Psychiatry.

[28] Osman M (2003). Therapeutic implications of sex differences in asthma and atopy. Arch Dis Child.

[29] Nafstad P, Magnus P, Jaakkola JJ (2000). Risk of childhood asthma and allergic rhinitis in relation to pregnancy complications. J Allergy Clin Immunol.

[30] Nafstad P, Nystad W, Magnus P, Jaakkola JJK. Asthma and allergic rhinitis at 4 years of age in relation to fish consumption in infancy. J Asthma. 2003;40:343–8. 10.1081/jas-120018633 12870829

[31] Wang X, Liu W, Hu Y, Zou Z, Shen L, Huang C (2016). Home environment, lifestyles behaviors, and rhinitis in childhood. Int J Hyg Environ Health.

[32] Spiekermann GM, Walker WA (2001). Oral tolerance and its role in clinical disease. J Pediatr Gastroenterol Nutr.

[33] Hanson LÅ (2007). Session 1: feeding and infant development breast-feeding and immune function. Proc Nutr Soc.

[34] Huang C, Liu W, Cai J, Weschler LB, Wang X, Hu Y (2017). Breastfeeding and timing of first dietary introduction in relation to childhood asthma, allergies, and airway diseases: a cross-sectional study. J Asthma..

[35] Norbäck D, Hashim JH, Markowicz P, Cai GH, Hashim Z, Ali F (2016). Endotoxin, ergosterol, muramic acid and fungal DNA in dust from schools in Johor Bahru, Malaysia - associations with rhinitis and sick building syndrome (SBS) in junior high school students. Sci Total Environ.

[36] Ng TP, Tan WC (1994). Epidemiology of allergic rhinitis and its associated risk-factors in Singapore. Int J Epidemiol.

[37] Bunnag C, Jareoncharsri P, Voraprayoon S, Kongpatanakul S (2000). Epidemiology of rhinitis in Thais : characteristics and risk factors. Asian Pacific J Allergy Immunol.

[38] Graif Y, Garty B-Z, Livne I, Green MS, Shohat T (2004). Prevalence and risk factors for allergic rhinitis and atopic eczema among schoolchildren in Israel: results from a national study. Ann Allergy Asthma Immunol.

[39] Zuraimi MS, Tham KW, Chew FT, Ooi PL, David K (2008). Home exposures to environmental tobacco smoke and allergic symptoms among young children in Singapore. Int Arch Allergy Immunol.

[40] Lei Y, Yang H, Zhen L (2016). Obesity is a risk factor for allergic rhinitis in children of Wuhan (China). Asia Pac Allergy.

[41] Norbäck D, Hashim JH, Cai GH, Hashim Z, Ali F, Bloom E (2016). Rhinitis, ocular, throat and dermal symptoms, headache and tiredness among students in schools from Johor Bahru, Malaysia: associations with fungal DNA and mycotoxins in classroom dust. PLoS One.

[42] Lee M-T, Wu C-C, Ou C-Y, Chang J-C, Liu C-A, Wang C-L (2017). A prospective birth cohort study of different risk factors for development of allergic diseases in offspring of non-atopic parents. Oncotarget.

[43] Yao TC, Ou LS, Yeh KW, Lee WI, Chen LC, Huang JL (2011). Associations of age, gender, and BMI with prevalence of allergic diseases in children: PATCH study. J Asthma.

[44] Kilpeläinen M, Terho EO, Helenius H, Koskenvuo M (2001). Home dampness, current allergic diseases, and respiratory infections among young adults. Thorax.

[45] Lee YL, Shaw CK, Su HJ, Lai JS, Ko YC, Huang SL (2003). Climate, traffic-related air pollutants and allergic rhinitis prevalence in middle-school children in Taiwan. Eur Respir J.

[46] Duggan EM, Sturley J, Fitzgerald AP, Perry IJ, Hourihane JOB (2012). The 2002-2007 trends of prevalence of asthma, allergic rhinitis and eczema in Irish schoolchildren. Pediatr Allergy Immunol.

[47] Gelber LE, Seltzer LH, Bouzoukis JK, Pollart SM, Chapman MD, Platts-Mills T a (1993). Sensitization and exposure to indoor allergens as risk factors for asthma among patients presenting to hospital. Am Rev Respir Dis.

[48] Dold S, Wjst M, von Mutius E, Reitmeir P, Stiepel E (1992). Genetic risk for asthma, allergic rhinitis, and atopic dermatitis. Arch Dis Child.

[49] Skoner DP (2001). Allergic rhinitis: definition, epidemiology, pathophysiology, detection, and diagnosis. J Allergy Clin Immunol.

[50] Helaskoski E, Suojalehto H, Virtanen H, Airaksinen L, Kuuliala O, Aalto-Korte K (2014). Occupational asthma, rhinitis, and contact urticaria caused by oxidative hair dyes in hairdressers. Ann Allergy Asthma Immunol.

[51] Pistiner M, Gold DR, Abdulkerim H, Hoffman E, Celedón JC (2008). Birth by cesarean section, allergic rhinitis, and allergic sensitization among children with a parental history of atopy. J Allergy Clin Immunol.

[52] Strachan DP (2000). Family size, infection and atopy: the first decade of the “hygiene hypothesis”. Thorax.

