# Supplementary material for: Epidemiology of allergic rhinitis and associated risk factors in Asia
Source: World Allergy Organ J. 2018 Aug 6;11(1):17. doi: 10.1186/s40413-018-0198-z (PMC6091170; doi:10.1186/s40413-018-0198-z)
Supplement: Supplementary file 1 — List of countries and dependent territories used in the literature review search. (PDF 322 kb) [file 40413_2018_198_MOESM1_ESM.pdf]

Additional file

Additional file 1: List of countries and dependent territories used in the literature review search

No.	Country (or dependent territory)	No.	Country (or dependent territory)	No.	Country (or dependent territory)
1	China	18	Malaysia	35	Kyrgyzstan
2	India	19	Nepal	36	Singapore
3	Indonesia	20	Afghanistan	37	Turkmenistan
4	Pakistan	21	Yemen	38	Palestine
5	Bangladesh	22	North Korea	39	Lebanon
6	Russia	23	Taiwan[6]	40	Oman
7	Japan	24	Sri Lanka	41	Kuwait
8	Philippines	25	Syria	42	Georgia
9	Vietnam	26	Kazakhstan	43	Mongolia
10	Iran	27	Cambodia	44	Armenia
11	Turkey	28	Azerbaijan	45	Qatar
12	Thailand	29	United Arab Emirates	46	Bahrain
13	Myanmar	30	Tajikistan	47	Timor-Leste
14	South Korea	31	Israel	48	Bhutan
15	Iraq	32	Hong Kong (China)	49	Macau (China)
16	Saudi Arabia	33	Jordan	50	Brunei
17	Uzbekistan	34	Laos	51	Maldives
